# Unraveling the gut: the pivotal role of intestinal mechanisms in Kawasaki disease pathogenesis

**DOI:** 10.3389/fimmu.2024.1496293

**Published:** 2024-11-26

**Authors:** Enfu Tao, Dandan Lang

**Affiliations:** ^1^ Department of Neonatology and Neonatal Intensive Care Unit (NICU), Wenling Maternal and Child Health Care Hospital, Wenling, Zhejiang, China; ^2^ Department of Pediatrics, Zhuhai People’s Hospital (Zhuhai Clinical Medical College of Jinan University), Zhuhai, Guangdong, China

**Keywords:** Kawasaki disease, gut permeability, systemic vasculitis, coronary artery aneurysms, intravenous immunoglobulin resistance, platelet activation, gut microbiota, short-chain fatty acids

## Abstract

Kawasaki disease (KD), an acute systemic vasculitis that primarily affects children under 5 years of age, is the leading cause of acquired heart disease in this age group. Recent studies propose a novel perspective on KD’s etiology, emphasizing the gastrointestinal (GI) tract, particularly the role of gut permeability. This review delves into how disruptions in gut barrier function trigger systemic inflammatory responses, exacerbate vascular inflammation, and contribute to coronary artery aneurysms. Evidence suggests that children with KD often exhibit increased gut permeability, leading to an imbalance in gut immunity and subsequent gut barrier damage. These changes impact vascular endothelial cells, promoting platelet aggregation and activation, thereby advancing severe vascular complications, including aneurysms. Additionally, this review highlights the correlation between GI symptoms and increased resistance to standard treatments like intravenous immunoglobulin (IVIG), indicating that GI involvement may predict therapeutic outcomes. Advocating for a new paradigm, this review calls for integrated research across gastroenterology, immunology, and cardiology to examine KD through the lens of GI health. The goal is to develop innovative therapeutic interventions targeting the intestinal barrier, potentially revolutionizing KD management and significantly improving patient outcomes.

## Introduction

Kawasaki disease (KD) is an acute febrile illness and systemic vasculitis that predominantly affects children under 5 years old (1). This condition frequently results in cardiovascular complications, including coronary artery lesions (CALs), coronary aneurysms, systemic artery aneurysms, and myocarditis. In addition to these cardiovascular issues, gastrointestinal (GI) symptoms such as abdominal pain, vomiting, and diarrhea are commonly observed, particularly in severe cases (2, 3). These manifestations can lead to significant long-term cardiovascular complications, such as vascular remodeling—characterized by the thickening and stiffening of blood vessel walls due to chronic inflammation—and myocardial fibrosis, involving the excessive accumulation of scar tissue in the heart muscle. These complications increase the risk of ischemic heart disease, myocardial infarction, and even death, making KD the leading cause of acquired heart disease in children in developed countries (4). A major complication associated with KD is the development of CAL. Without timely treatment, approximately 25% of children with KD may develop CAL, significantly increasing the risk of long-term cardiovascular issues (5). However, treatment with high-dose intravenous immunoglobulin (IVIG) combined with aspirin can significantly reduce this risk (6). Despite these measures, 10%–20% of patients exhibit resistance to IVIG, leading to an increased risk of developing coronary artery aneurysms (5). These data underscore the critical need for early diagnosis and effective treatment strategies to mitigate severe cardiac outcomes in KD.

Recently, the GI manifestations of KD have attracted increasing scholarly attention as potentially significant contributors to the disease’s etiology. A notable multicenter retrospective study from Italy reported that 35% of KD patients presented with GI symptoms, which were independently associated with a higher risk of IVIG resistance and the development of coronary artery aneurysms (3). When GI symptoms precede the hallmark clinical features of KD, diagnosis and treatment may be delayed, potentially leading to unnecessary surgical interventions and a heightened risk of CAL (2, 3). This suggests that GI manifestations might not merely be a secondary symptom but could play a pivotal role in influencing KD’s clinical course.

While the precise cause of KD remains unclear, it likely originates from an aberrant immune response to environmental triggers in genetically susceptible individuals (7, 8). Key immune cells involved in KD pathogenesis include monocytes, platelets (PLT) (9), macrophages (10), neutrophils (11), CD8^+^ T cells (12), and antigen-specific IgA plasma cells (13). Monocytes and macrophages are early responders, producing proinflammatory cytokines like tumor necrosis factor (TNF)-α, interleukin (IL)-1, and IL-6, which amplify inflammation and contribute to vascular damage (10, 14). Activated PLT form aggregates with immune cells like monocytes, enhancing the release of proinflammatory cytokines such as IL-1β, contributing to the development of CAL and disease severity (9). Neutrophils, as the initial responders in acute inflammation, generate reactive oxygen species (ROS) that can damage the endothelium and stimulate the release of proinflammatory cytokines like IL-1β and TNF (11). CD8^+^ T cells are key contributors to the development of coronary arteritis in KD, infiltrating coronary tissues and driving inflammation that leads to vascular damage (12). These cellular elements are crucial in activating several signaling pathways that contribute to KD’s pathology. Notable among these are the CD40–CD40L signaling pathway (15), the interleukin (IL)-33–suppression of tumorigenicity 2 (ST2) axis (16), and the nucleotide-binding domain, leucine-rich repeat, and pyrin domain-containing protein 3 (NLRP3) inflammasome–IL-1 pathway (17), all of which play vital roles in the inflammatory and immune responses characteristic of KD.

As research into KD progresses, it is becoming increasingly evident that the interactions among the cardiovascular, immune, and GI systems are complex yet critical in determining the disease’s clinical trajectory. The recent recognition of GI manifestations as potential drivers of disease progression and therapeutic response opens new pathways for research and clinical intervention. This review aims to consolidate and analyze existing knowledge concerning the role of the GI tract in KD. By examining the intricate interactions within the GI system that may influence the pathogenesis of KD, it seeks to identify gaps in the current research and propose areas for future investigation. A deeper understanding of these mechanisms could contribute to the development of more effective diagnostic tools, targeted therapies, and comprehensive management strategies, ultimately improving patient outcomes in KD. This approach aligns with the broader objective of advancing the understanding and treatment methodologies for this condition.

## The association of GI symptoms and Kawasaki disease outcomes

Early recognition of GI symptoms in KD is crucial, as they often indicate a more severe disease course, with a higher likelihood of resistance to IVIG therapy and an increased incidence of CAL (2, 3). A multicenter retrospective study in Italy reported that 35% of children with KD displayed GI symptoms, which were identified as independent risk factors for IVIG resistance and the development of coronary artery aneurysms (3). Moreover, the early presentation of GI symptoms, rather than the characteristic clinical features of the disease, may delay diagnosis and treatment, potentially leading to unnecessary surgical interventions and an increased risk of CAL (2, 3). Furthermore, in a comprehensive domestic study involving 1,490 children with KD, patients were categorized into three groups based on the early appearance of GI symptoms relative to the typical KD phenotype. The study revealed that the rate of IVIG resistance was significantly higher in the GI symptom group compared to the control group, highlighting GI symptoms as a risk factor for IVIG unresponsiveness and CAL (18). Most recently, Huang et al. showed that children presenting with jaundice as a predominant symptom of KD have a higher risk of IVIG-refractory disease, CAL, and more frequent recurrence of KD symptoms (19). The pathophysiology of jaundice in KD remains unclear but is thought to involve several factors, including vasculitis of the hepatobiliary vessels, cystic duct wall edema, inflammation of the liver and gallbladder serosa, and cholangitis (19, 20). Notably, 58.3% of these patients also presented with GI symptoms, suggesting that GI mechanisms might play a significant role in the development of coronary artery aneurysms and IVIG resistance (19). In addition, some researchers propose that the GI tract may be the primary site of onset for KD (21). However, the role of the GI tract in the pathogenesis of KD and its related molecular mechanisms warrants further research. The association between GI symptoms and KD outcomes is summarized in **Table 1**.

**Table 1:** Association between gastrointestinal symptoms and outcomes in patients with Kawasaki disease.

Published year	Incidence of GI symptoms	Types of GI symptoms	KD outcomes	References
2018	106/302 (35.1%)	Vomiting, diarrhea, abdominal pain, abdominal distension, paralytic ileus, jaundice, pancreatitis, and pseudo-obstruction (NA)	IVIG-resistant: 21/106 (19.8%), coronary ectasia: 17/106 (16.0%), coronary aneurysms: 10/106 (9.4%)	(3)
2018	NA	Abdominal pain: 34/49 (69%), vomiting: 24/49 (49%), diarrhea: 14/49 (29%)	Pericardial effusion: 3/49 (6.1%), artery abnormality: 21/49 (42.9%)	(2)
2013	7/118 (6%)	Vomiting: 5/7 (71.4%), diarrhea: 4/7 (57.1%), abdominal pain: 7/7 (100%), clinical jaundice: 3/7 (42.9%)	IVIG-refractory: 2/7 (28.6%), artery abnormality: 1/7 (14.3%)	(22)
2023	141/1,490 (9.46%)	Diarrhea: 100/141 (70.9%), vomiting: 55/141 (39.0%), abdominal pain: 34/141 (24.1%)	IVIG unresponsive: 26/141 (18.4%), artery abnormality: 28/141 (19.9%)	(18)
2024	6/12 (50%)^a^	Vomiting: 5/12 (41.7%), diarrhea: 4/12 (33.3%)	IVIG-refractory: 3/6 (50%), coronary artery abnormality: 4/6 (66.7%)	(19)
2005	10/219 (4.6%)	Abdominal pain: 10/10 (100%), abdominal distension: 7/10 (70%), vomiting and hepatomegaly: 6/10 (60%), diarrhea: 3/10 (30%), hematemesis: 1/10 (10%)	Coronary artery abnormality: 5/10 (50%)	(21)

NA, not available; GI, gastrointestinal.

a Jaundice-predominant manifestation of Kawasaki disease.

## Structural and functional components of the gut barrier

The intestinal barrier plays a vital role in maintaining overall health by regulating the entry of substances from the gut into the bloodstream. It selectively permits essential nutrients while blocking harmful agents and pathogens. This barrier’s protective function is achieved through three key components: the mucus layer, epithelial barrier, and gut vascular barrier (GVB) (23). The mucus layer is a protective gel-like coating that lines the intestines, preventing harmful substances and pathogens from reaching the epithelial cells (24). The epithelial barrier consists of tightly connected cells that defend against potential threats in the gut (25). Finally, the GVB regulates the movement of substances between the bloodstream and the gut, ensuring that only essential nutrients are absorbed while harmful substances are blocked (26). Together, these components facilitate the absorption of essential nutrients while preventing harmful substances and pathogens from entering the bloodstream. This helps protect the host physiology and regulate gut functions (27).

### Mucus barrier

The mucus barrier is a critical component of the intestinal defense system, acting as the first line of protection against pathogens and harmful substances. It shields the intestinal lining from physical and microbial threats while supporting a balanced gut microbiome and regulating immune responses. Specialized goblet cells in the intestinal lining produce mucins, which are glycoproteins that form the foundation of this protective layer. This mucus not only traps pathogens—preventing them from reaching epithelial cells—but also creates a dynamic ecosystem that maintains gut health (28). By separating pathogens from the intestinal lining, the mucus barrier protects the gut from infection and inflammation (29). Additionally, it reduces friction and mechanical stress within the digestive tract. Goblet cells play a role in immune regulation by presenting antigens from the gut lumen to the immune system, thereby facilitating adaptive immune responses (28). The mucus layer is primarily composed of materials secreted from the epithelium. Core components produced by goblet cells include mucin-2 (MUC2), the mucus-associated protein FCGBP (IgGFc-binding protein), and calcium-activated chloride channel regulator 1 (CLCA1) (30, 31). MUC2 is the main glycoprotein, forming the structural backbone of the mucus layer and serving as a crucial element in the innate defense against enteric infections (32). Impairments in MUC2 expression or function can result in a thinner or compromised mucus layer, making the intestine more vulnerable to pathogens and toxins, which may lead to inflammation and other gut-related diseases (33). FCGBP binds to MUC2, helping to stabilize the mucus layer and maintain its structural integrity (34). CLCA1 is a metalloprotease and a crucial non-mucin component of the mucus. It cleaves MUC2, significantly contributing to the regulation of the intestinal mucus layer’s stability and dynamics (31).

### Intestinal epithelial barrier

Beneath the mucus layer lies the intestinal epithelial barrier, a semipermeable physical and biochemical shield that plays a crucial role in maintaining gut homeostasis. This barrier facilitates a finely balanced interaction and separation between the gut microbiota and the host, allowing for the absorption of essential nutrients while preventing harmful substances, such as toxins and pathogens, from crossing into the bloodstream (27). Its functionality is attributed to the diverse types of epithelial cells it comprises, including predominant absorptive enterocytes, along with specialized cells such as enterochromaffin cells, goblet cells, Paneth cells, tuft cells, M cells, and stem cells (35, 36). Enterocytes are specialized cells that line the intestines, playing a crucial role in nutrient absorption. These cells are the primary absorptive units of the gut, responsible for taking in nutrients and transporting them into the bloodstream. In addition to absorption, enterocytes contribute to the intestinal barrier by forming a tightly connected layer that prevents harmful substances from crossing into the body. The epithelial layer is structured with four sets of intercellular junctions: tight junctions (TJ) [zonula occludens (ZO)], adherens junctions (zonula adherens), desmosomes, and gap junctions. These junctions collectively form the apical junctional complex, which is essential for regulating epithelial barrier function and intercellular transport (37, 38). TJ are structures that seal the gaps between enterocytes, acting as gatekeepers to regulate the passage of substances and maintain the selective permeability of the intestinal lining. They are essential for preserving the integrity and impermeability of the gut barrier (39). The formation of TJ is mediated by three primary types of transmembrane proteins: the claudin protein family (e.g., claudin-1), TJ-associated MARVEL proteins (TAMPs, such as occludin), and immunoglobulin-like cell adhesion molecules (e.g., junctional adhesion molecule A, JAM-A). Transmembrane proteins span the cell membrane and help anchor adjacent cells together, ensuring that the barrier remains strong and intact (40). Adherens junctions maintain cell-to-cell adhesion by connecting the actin cytoskeletons of neighboring cells through cadherin proteins. They provide structural support to the epithelial barrier, ensuring tissue integrity and facilitating cellular communication (41). Desmosomes are essential for maintaining the mechanical stability and integrity of the intestinal epithelium. They link intermediate filaments between neighboring cells, allowing the epithelium to endure physical stress and preserve the structural integrity of the gut lining under various conditions (42). Gap junctions are specialized intercellular connections that facilitate communication between adjacent cells by allowing the transfer of ions and small molecules. In the intestinal barrier, gap junctions contribute to the coordination of cellular activities, maintaining the integrity of the epithelium and regulating immune responses, which are crucial for maintaining homeostasis and barrier function (43). Disruption of these junctions can lead to increased intestinal permeability, commonly referred to as a “leaky gut,” a condition associated with various inflammatory diseases (44).

Transport mechanisms across the gut epithelium are complex, incorporating transcellular routes such as passive diffusion, receptor-mediated transport, vesicular transport or endocytosis, and paracellular transport, which includes both “pore” and “leak” pathways (27, 37, 45). These pathways are meticulously regulated, especially those involving TJ. Conversely, the “unrestricted” pathway comes into play when there are gaps in the epithelial barrier due to cell damage or death (46). Additionally, the gut barrier contains ATP-binding cassette (ABC) transporters which help avoid toxin accumulation and excessive inflammation by expelling unwanted substances (47).

### Gut vascular barrier

The GVB is a recently recognized and crucial component of the intestinal defense system, situated beneath the intestinal epithelium. It acts as the innermost protective layer, playing a key role in regulating the movement of substances between the gut and the bloodstream (26, 48). The GVB maintains selective permeability, allowing only small molecules such as water, electrolytes, nutrients, vitamins, minerals, gases, metabolites, hormones, and small peptides to pass through under physiological conditions. However, it blocks large molecules (greater than 4 kDa) and various harmful substances, including microbiota and their products, from entering the bloodstream (26). This barrier function helps prevent these substances from reaching the portal circulation and liver, thereby protecting systemic health (49, 50). The structural integrity of the GVB is crucial for regulating the gut–liver axis and controlling systemic inflammation (49, 50). It is comprised of a monolayer of endothelial cells, sealed by adherent and tight junctions and supported by pericytes and enteric glial cells. The fenestrated nature of the endothelial lining—characterized by small pores or “windows” known as fenestrae—facilitates the barrier’s selective permeability. These fenestrae are surrounded by plasmalemmal vesicle protein 1 (PV-1), which regulates its permeability (45, 51). Pericytes are specialized cells that wrap around the endothelial cells, contributing to the stability of blood vessels and regulating blood flow (48). Meanwhile, enteric glial cells are critical for maintaining the integrity of the GVB. Experiments in transgenic mice show that loss of enteric glial cells leads to dysfunction of the intestinal mucosal barrier and subsequent inflammation (52). Additionally, the integrity of the GVB is supported by the Wnt/beta-catenin signaling pathway, which plays a key role in the maintenance of endothelial cells and overall barrier function (53).

Disruptions of the GVB have been implicated in various conditions including liver disease, colorectal cancer, inflammatory bowel diseases (IBD), celiac disease, ankylosing spondylitis, and gut-derived sepsis (51, 54–56). Additionally, in cases of neonatal meningitis, the immaturity of the neonatal intestinal microbiota contributes to increased susceptibility to group B *Streptococcus* (GBS) intestinal colonization and heightened permeability of the GVB, suggesting that both disruption and immaturity of the GVB can be pathogenic (57).

### Gut microbiota

Intestinal microbes, collectively known as the gut microbiota, play a crucial role in maintaining human health. These microbes support gut barrier function, protect against harmful pathogens, aid in nutrient synthesis and digestion, and enhance the development of the host’s innate and adaptive immune systems (58, 59). While the gut microbiota does not form a physical barrier like the mucus layer or the intestinal epithelium, it functions as a critical line of defense within the GI tract. The symbiotic relationship between the gut microbiota and the gut barrier exemplifies a significant evolutionary adaptation, where microbes not only reinforce barrier integrity but also shape immune responses and impact overall health (45).

The gut microbiota establishes crucial signaling connections with the mucus barrier, intestinal epithelial barrier, and GVB. It influences the mucus barrier by regulating the synthesis and glycosylation of mucin, particularly MUC2, the primary mucus component. Mucin is a glycoprotein that makes up the mucus, providing a protective layer over the gut lining. This regulation modifies the mucus’ viscosity and functionality, enhancing its role as a protective barrier (60). This mutualistic relationship benefits both the human gut and the microbes it hosts. The gut provides a suitable environment for microbes, and in return, these microbes regulate important physiological functions, such as mucin production (61). The composition and abundance of intestinal bacteria affect the production, expansion, and degradation of mucus, as well as goblet cell numbers, underscoring the critical role of microbe–host interactions in maintaining mucosal barrier integrity (62). In addition, bacterial components and metabolites significantly influence MUC2 production and maturation. For example, lipopolysaccharide (LPS), a component of Gram-negative bacterial membranes, is a key factor in triggering low-grade systemic inflammation due to its strong proinflammatory properties (37). When LPS binds to Toll-like receptor (TLR) 4 on immune cells, it initiates a myeloid differentiation primary response 88 (MyD88)-dependent signaling cascade, activating nuclear factor kappa light-chain enhancer of activated B cells (NF-κB). This cascade results in the production of pro-inflammatory cytokines, including IL-1β, IL-6, IL-8, and TNF-α, which play essential roles in immune responses (63). Additionally, LPS-induced cytokines like TNF-α and IFN-γ can impair TJ’s integrity by decreasing the expression of crucial proteins such as occludin, claudin, and ZO-1; increasing gut permeability; and weakening the gut barrier (44). Furthermore, LPS activates the TLR/MyD88/NF-κB/NOD-like receptor family pyrin domain-containing 6 (NLRP6) inflammasome signaling pathway, inducing a protective hypersecretion of MUC2 by goblet cells in response to infections (64).

Another group of microbial metabolites, short-chain fatty acids (SCFAs)—such as butyrate, propionate, and acetate—is produced by bacterial fermentation of dietary fibers. These SCFAs are essential for maintaining gut health as they serve as a key energy source for intestinal epithelial cells (IECs) and regulate MUC2 gene transcription, influencing mucus production (65). In addition, SCFAs strengthen the intestinal barrier, primarily by modulating TJ proteins, which are essential for epithelial integrity. Butyrate, in particular, activates AMP-activated protein kinase (AMPK), promoting the assembly of TJ proteins and enhancing barrier function (66). Research by Feng et al. demonstrated that SCFAs like acetate, propionate, and butyrate improve the expression of TJ proteins in IECs, supporting the maintenance of key components like ZO-1 and occludin. These treatments also enhance paracellular transport, inhibit the activation of the NLRP3 inflammasome, and promote autophagy, further solidifying the gut barrier (67).

**Figure 1:**
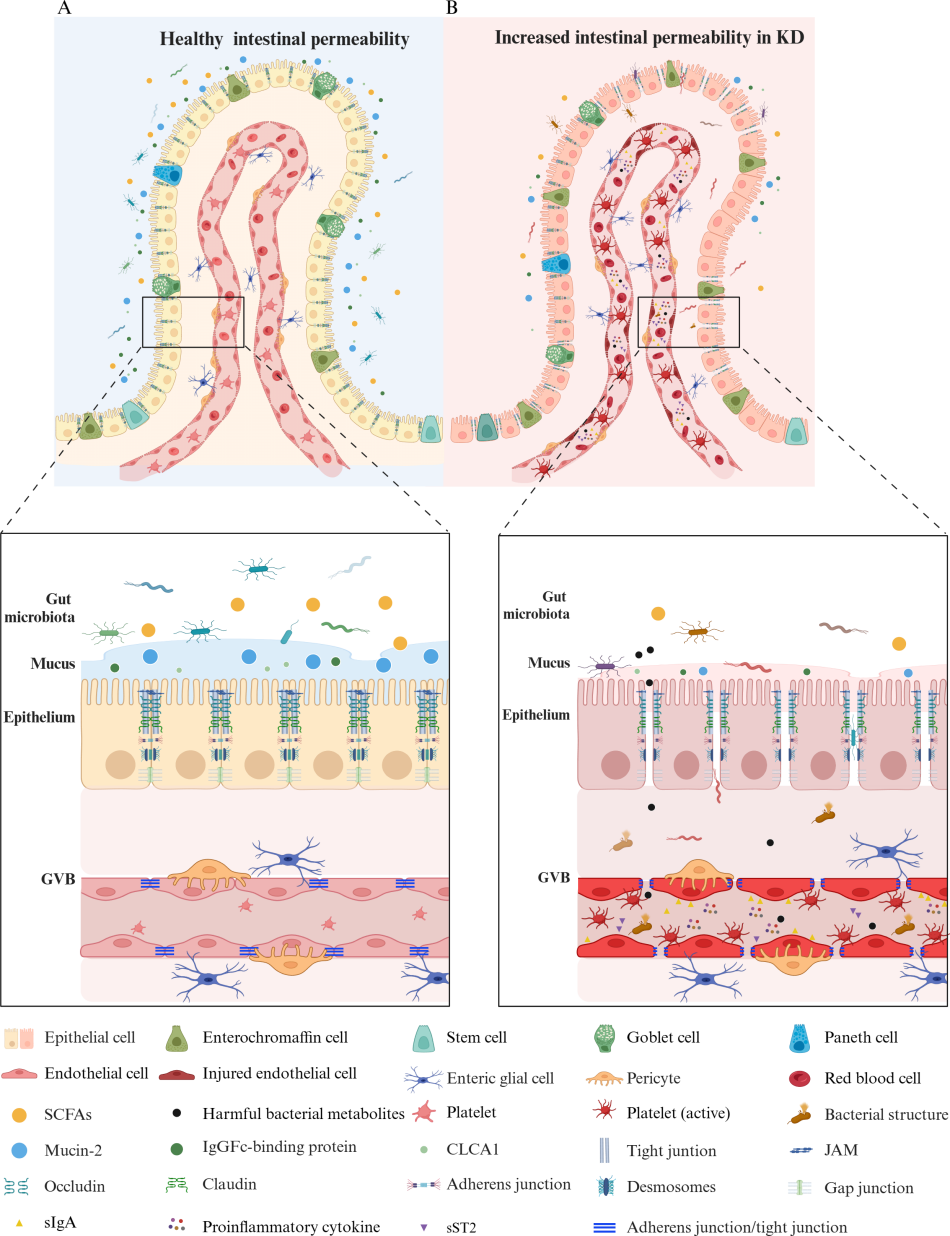
A Comparison of gut permeability in healthy vs. Kawasaki disease states. **(A)** Healthy gut permeability. In a healthy state, a balanced gut microbiota supports a mucus layer, rich in mucin-2 produced by goblet cells, that serves as a protective barrier. This layer, primarily composed of mucin-2, FCGBP, and CLCA1, traps pathogens, stabilizes its structure, and adjusts thickness to safeguard gut health and maintain microbiome balance. The epithelial cells—including enterocytes, enterochromaffin cells, Paneth cells, goblet cells, and stem cells—are tightly connected by junctional complexes like tight junctions (claudin, occludin, ZO-1), adherens junctions, desmosomes, and gap junctions, ensuring selective permeability. The GVB remains intact, with endothelial cells linked by adherens and tight junctions, supported by pericytes and enteric glial cells. **(B)** Increased gut permeability in Kawasaki disease. In KD, the gut barrier is compromised, showing an imbalance in the microbiota with decreased SCFAs-producing bacteria and an increased presence of harmful bacterial structures and metabolites. The mucus layer is degraded, reducing mucin-2, FCGBP, and CLCA1, weakening the first line of defense. Junctional complexes such as tight junctions, adherens junctions, and desmosomes are disrupted, increasing permeability. The compromised GVB exhibits injured endothelial cells with weakened connections, allowing for the translocation of pathogens, inflammatory mediators, and platelets into the bloodstream. Active platelets and proinflammatory cytokines (TNF-α, IFN-γ, IL-1β, IL-6) increase in response to gut barrier disruption, contributing to systemic inflammation. Elevated sST2 and sIgA in the bloodstream further indicate barrier dysfunction and inflammatory activity. KD, Kawasaki disease; GVB, gut vascular barrier; SCFAs, short-chain fatty acids; sIgA, secretory immunoglobulin A; MUC2, mucin-2; FCGBP, IgGFc-binding protein; CLCA1, calcium-activated chloride channel regulator 1; ZO-1, zonula occludens 1; JAM, junctional adhesion molecule; TNF, tumor necrosis factor; IFN, interferon; IL, interleukin; sST2, soluble ST2. The figure has been revised and adapted from Brescia et al. (48).

In summary, the gut barrier is an intricate system, pivotal in regulating host health and GI homeostasis. The gut permeability in a healthy state is displayed in **Figure 1A**.

## Increased gut permeability: a gateway to inflammation in Kawasaki disease

The exact etiology of KD remains unclear (8). Currently, KD is thought to be triggered by infectious agents interacting with genetic and environmental factors, leading to an abnormal immune response characterized by elevated inflammation in the acute phase (7, 68). Although infections are acknowledged as a significant factor in KD onset, the exact pathogens responsible remain unidentified (7). This review explores a critical pathway—the enhancement of gut permeability and its role as a catalyst for inflammatory responses in KD. The focus on gut permeability stems from recent research revealing its intricate interactions with the immune system, which may contribute to KD progression (69, 70). The following sections will discuss how impairments in gut barrier function could allow pathogens to enter the bloodstream from the gut, thereby initiating systemic inflammatory responses in individuals with KD.

The gut epithelial barrier serves as the body’s first line of defense, providing a separation from the external environment while facilitating nutrient absorption (27). This barrier is strengthened by microbial communities and their metabolites, along with structural and functional elements such as tight junctions, antimicrobial peptides, secretory immunoglobulin A (sIgA), and a protective mucus layer (27, 71). Together, these components foster a symbiotic relationship between the host and its microbiota, ensuring a stable and healthy gut environment (71). An increasing number of clinical and preclinical studies have identified impairments in intestinal epithelial barrier function in KD (1, 70, 72, 73). The direct consequence of gut barrier dysfunction is an increase in gut permeability (37). Factors impacting barrier function, such as imbalances in gut microbiota, changes in the expression of intercellular junction proteins (27), and decreased sIgA, can increase gut permeability (27, 74). Specifically, microbial dysbiosis can disrupt the composition of gut-resident communities, leading to changes in microbial metabolites, which impact the integrity of the gut barrier through modulation of TJ proteins and immune responses. This disruption often results in increased paracellular permeability, facilitating the translocation of antigens and triggering inflammatory pathways (27). sIgA plays a crucial role in maintaining gut barrier integrity by preventing the adherence and invasion of pathogens. It helps in immune exclusion by binding to microorganisms and toxins, thereby inhibiting their contact with epithelial cells. A decrease in sIgA can compromise this barrier function, leading to increased gut permeability and a higher risk of inflammation. The presence of sIgA is vital in sustaining a balanced interaction between the host and the gut microbiota, contributing to overall intestinal homeostasis (74). Recent studies have consistently highlighted the role of gut microbiota in KD, indicating a pattern of dysbiosis that could contribute to disease susceptibility and progression. Teramoto et al. demonstrated that dysbiosis—marked by an increase in proinflammatory *Ruminococcus gnavus* and a decrease in inflammation-suppressing *Blautia*—could heighten susceptibility to KD by affecting microbial diversity and inflammatory responses (68). Similarly, metagenomic analysis of Kinumaki et al. on 28 KD patients revealed a significant presence of *Streptococcus* spp. during the acute phase and *Ruminococcus* during the non-acute phase, suggesting specific bacterial roles in the disease’s progression (75). In alignment with these findings, Shen et al. found significant dysbiosis in children with acute KD, particularly a reduction in *Bacteroidetes* and *Dorea*—bacterial groups associated with healthy gut function—further linking altered microbiota to KD’s pathogenesis (76). Expanding on these insights, Chen et al. linked gut microbiota alterations with systemic inflammation in children with KD, noting that decreased fecal microbial diversity and increased inflammatory markers like IL-6 associated with specific bacteria such as *Enterococcus* and *Helicobacter* highlighting their potential role in exacerbating inflammatory responses in KD (77). Together, these studies provide a cohesive view of how shifts in gut microbiota—both in diversity and specific bacterial populations—might underpin KD’s immune dysfunction, warranting further exploration of gut-based interventions to mitigate disease severity. Although these studies primarily focus on gut microbiota alterations and their association with systemic inflammation in KD, they suggest a broader implication. Microbial dysbiosis can disrupt the composition of gut-resident communities, leading to changes in microbial metabolites. These alterations are known to affect the integrity of the gut barrier by modulating TJ proteins and influencing immune responses. Therefore, while these studies do not explicitly investigate gut permeability, their findings highlight a potential pathway through which microbiota-driven changes could compromise the gut barrier in KD. This calls for future research to examine whether the observed dysbiosis in KD patients is linked to increased gut permeability and, consequently, to systemic inflammatory responses. Building on the observed microbial imbalances, Kaneko et al. proposed that dysbiosis—characterized by reduced butyrate-producing bacteria—could contribute to KD’s etiology by disrupting the balance of T helper 17 (Th17) cells and regulatory T (Treg) cells, potentially triggering hypercytokinemia (7). Recently, a comprehensive retrospective case–control study encompassing 17,818 KD patients and 89,090 matched control subjects revealed that antibiotic usage within the past 6 or 12 months is linked to KD. Moreover, this association becomes more pronounced as the variety of antibiotic classes used increases (78). Taken together, these studies suggest that dysbiosis of the gut microbiota may be linked to the pathogenesis of KD. However, a critical methodological issue in recent studies is that fecal samples from KD patients are often collected after antibiotic treatments, making it difficult to distinguish microbiota changes due to KD from those caused by antibiotics. In the diagnosis of KD, antibiotics are typically administered before a definitive diagnosis is made, especially in atypical cases, which relies on meeting five clinical criteria (79, 80). Consequently, changes in the gut microbiota detected by 16S rRNA or metagenomic sequencing might not truly represent the pathogenesis of KD but instead reflect the effects of antibiotics. This underscores the importance of prospective studies that collect fecal samples from febrile patients before diagnosis, providing a clearer understanding of the actual microbiota alterations in KD, separate from antibiotic influence.

Research indicates that patients with KD exhibit increased gut permeability, suggesting a compromised gut barrier that allows pathogens and other harmful substances to translocate into the bloodstream, triggering immune responses and potentially contributing to systemic inflammation and vascular damage (70, 73). This is evidenced by heightened serum concentrations of sIgA, indicative of impaired gut barrier function (3, 72). Changes in the gut microbiota, including a decrease in SCFAs-producing bacteria, are linked to increased permeability (81, 82) and inflammation in KD (1). Wang et al. demonstrated in a KD mouse model that a decline in SCFAs-producing bacteria led to reduced expression of key gut barrier proteins—such as claudin-1, JAM-1, occludin, and ZO-1—and higher plasma D-lactate levels, intensifying inflammation. However, administration of the probiotic *Clostridium butyricum* improved barrier function by boosting SCFAs production (1). Furthermore, clinical studies showed that fecal butyrate concentrations were significantly lower in KD patients compared to health control although the sample size was limited (*n* = 4 for both groups) (7). These data suggest that reduced SCFAs levels may be linked to increased gut permeability in children with KD. SCFAs fortify the gut barrier by enhancing the mucus layer that shields the IECs from pathogens, primarily through the secretion of mucins and antimicrobial peptides, which are crucial for both microbial and chemical barriers (83). Furthermore, SCFAs promote barrier stability by encouraging a balanced microbial environment, inhibiting harmful pathogen colonization while supporting beneficial microbiota growth. This activity driven by the secretion of mucins and antimicrobial peptides bolsters both microbial and chemical barriers, enhancing the protective mucus layer over the IECs (83). SCFAs also regulate inflammatory processes by controlling innate immune sensors such as TLR and NLRP3 inflammasomes, thereby mitigating excessive inflammatory signaling (84). Consequently, reduced SCFAs levels may lead to increased gut permeability, allowing pathogens and toxins to enter the bloodstream, triggering abnormal activation of subepithelial immune cells and overproduction of inflammatory mediators like TNF-α, IFN-γ, and IL-1β, which may contribute to the pathogenesis of KD (82, 85). IL-1β, for instance, increases IECs TJ permeability by activating signaling pathways involving NF-κB, upregulating the myosin light-chain kinase gene, and post-transcriptionally regulating the occludin gene via microRNA, thereby influencing intestinal inflammation (86). IL-1 signaling plays a crucial role in KD’s pathogenesis, as IL-1α and IL-1β, once activated, create a local proinflammatory environment, prompting vasodilation and recruiting monocytes and neutrophils to damaged tissue sites. Both IL-1α and IL-1β have been implicated in myocarditis and aneurysm formation in the *Lactobacillus casei* cell-wall extract (LCWE) mouse model of KD (87). Moreover, IL-1 signaling in IECs increases intestinal permeability, while deleting IL-1R specifically in these cells prevents permeability increases and reduces heart inflammation and abdominal aorta aneurysms in LCWE-injected KD mice (70). These findings highlight the need for further research on increased gut permeability in children with KD, as well as the role of SCFAs levels, gut microbiome imbalances, and their link to the disease. Early intervention to correct gut permeability and restore gut microbiome balance or SCFAs levels may offer promising strategies for treating or preventing KD. A comparative hypothesis of gut permeability in a healthy state versus KD is depicted in **Figures 1A, B**.

## Gut permeability and liver damage in Kawasaki disease: exploring the gut–liver axis

**Figure 2:**
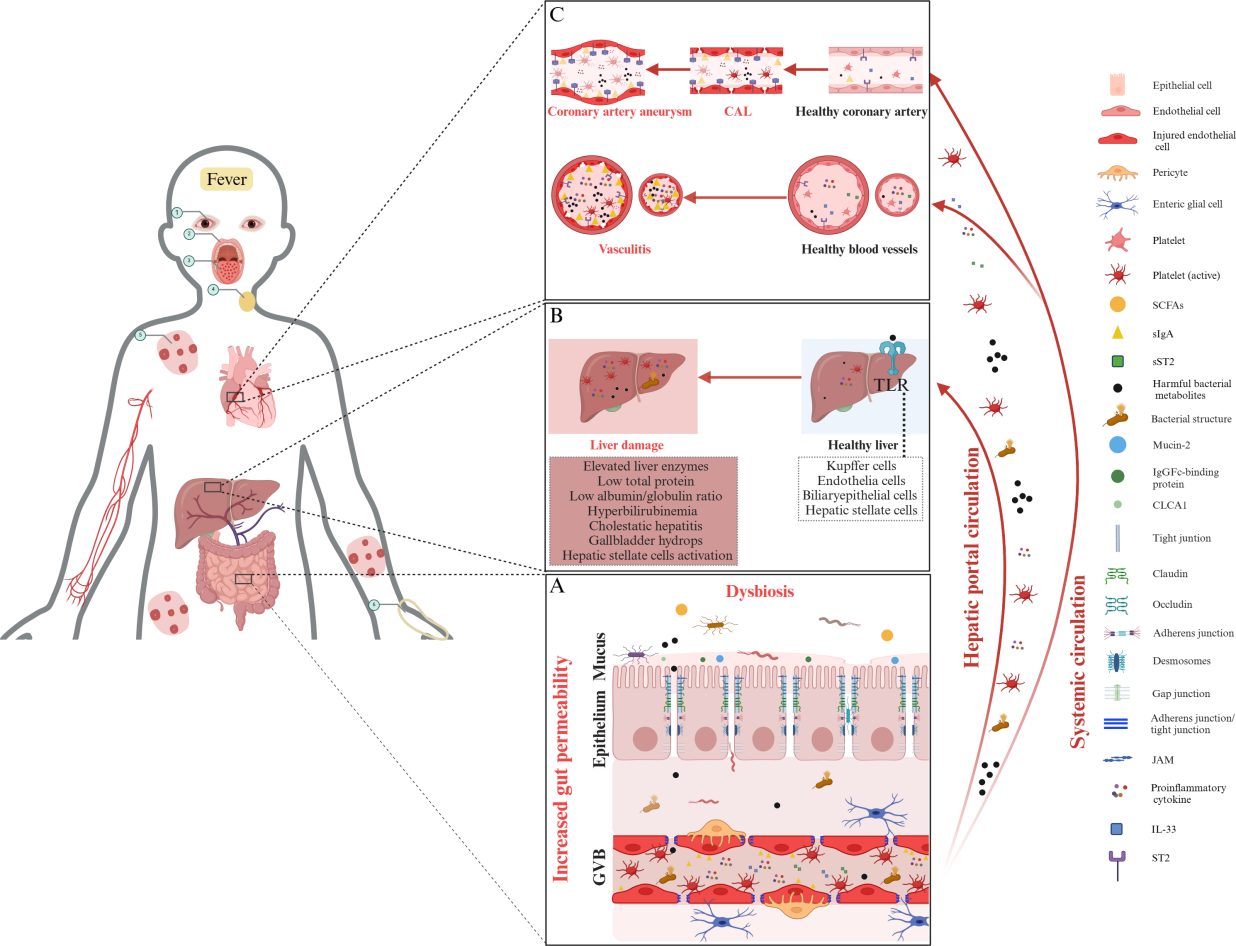
The role of gut permeability and systemic inflammation in Kawasaki disease pathogenesis. **(A)** Increased permeability and dysbiosis in KD. In KD, gut barrier dysfunction, characterized by dysbiosis, a compromised mucus layer, weakened epithelial tight junctions, and damaged GVB, allows harmful bacterial metabolites and structures to enter the bloodstream, initiating systemic inflammation. **(B)** The gut–liver axis and liver damage. Increased gut permeability facilitates the translocation of harmful bacterial metabolites, microbial structures, inflammatory cytokines, and activated platelets into the liver through the hepatic portal circulation. This translocation can activate Toll-like receptors in hepatic tissue, potentially leading to liver dysfunction. Common manifestations include elevated liver enzyme levels, decreased total protein, and clinical presentations such as cholestatic hepatitis, gallbladder hydrops, and hepatic stellate cell activation. **(C)** Hypothesis linking gut permeability to vasculitis and coronary artery lesions in KD. Increased gut permeability allows for the translocation of harmful bacterial metabolites, bacterial structures, and proinflammatory cytokines into systemic circulation. Upon reaching coronary arteries, these elements can trigger vasculitis and CAL, leading to the formation of coronary artery aneurysms. A key element in this inflammatory process is the elevated level of IL-33, which interacts with its receptor, ST2, exacerbating inflammation. ST2 exists in two forms: ST2L, which promotes inflammation when IL-33 binds to it; and sST2, which acts as a decoy receptor, binding IL-33 in the bloodstream to regulate its activity. The IL-33/ST2 signaling axis, particularly the balance between ST2L and sST2, is hypothesized to play a significant role in the initiation of vasculitis and the progression of CAL in KD. Moreover, elevated gut permeability allows sIgA to translocate into the bloodstream, where it may deposit in coronary artery tissues. This accumulation of sIgA in vascular walls activates local immune responses, contributing to inflammation and tissue damage, and is thought to play a significant role in the development of vasculitis and CAL associated with KD. The circled Arabic numerals from 1 to 6 depict the clinical signs of KD, including conjunctivitis, cracked lips, “strawberry” tongue, swollen cervical lymph nodes, polymorphous rash, and edema of the hands, respectively. Note: Proinflammatory cytokines include tumor necrosis factor (TNF)-α, interferon (IFN)-γ, interleukin (IL)-1β, and IL-6. KD, Kawasaki disease; SCFAs, short-chain fatty acids; CAL, coronary artery lesion; sIgA, secretory immunoglobulin A; ST2, serum stimulation 2; sSt2, soluble ST2; CLCA1, calcium-activated chloride channel regulator 1; GVB, gut vascular barrier; JAM, junctional adhesion molecule; TLR, Toll-like receptor. The figure has been revised and adapted from Brescia et al. (48).

The increase in gut permeability observed in KD patients may be intricately linked to liver damage. Due to the liver’s proximity to the GI tract, metabolites from the gut microbiota can directly enter the liver through the portal vein, potentially impacting liver function (48). This relationship is central to the gut–liver axis, which describes how gut microbiota and their by-products impact liver function and the progression of liver diseases (88). Normally, an intact gut barrier protects the body and the portal circulation from harmful substances. However, a compromised barrier allows microorganisms and their by-products to migrate to the liver via the portal vein (89, 90). These gut-derived bacterial products activate TLR on various liver cells—such as Kupffer cells, endothelial cells, dendritic cells, and hepatocytes—contributing to both acute and chronic liver conditions (90). Liver dysfunction is common in children with KD, ranging from mild enzyme elevation to severe cholestatic hepatitis or gallbladder hydrops (18). A study of 210 KD patients found that 90.95% had abnormal liver function test (LFT), with hypoalbuminemia being the most common, followed by elevated aspartate aminotransferase (AST), reduced total protein (TP), low albumin/globulin (A/G) ratio, and hyperbilirubinemia (91). Furthermore, meta-analyses and retrospective cohort studies have linked liver abnormalities with increased IVIG resistance in patients with KD (92, 93). For instance, a retrospective study of 1,490 KD patients identified GI involvement, prolonged fever, elevated alanine aminotransferase (ALT), PLT count, and C-reactive protein (CRP) as risk factors for CAL. Those with liver dysfunction had longer hospital stays, higher IVIG resistance and an increased incidence of CAL, and elevated CRP, suggesting that liver function may reflect the severity of systemic inflammation (18). Conversely, a study of 259 patients found no significant difference in CAL prevalence between those with normal and abnormal LFT, possibly due to a smaller sample size (94). Despite substantial evidence suggesting a potential link between gut permeability and liver dysfunction in KD, direct research is limited, representing a knowledge gap. Future studies should investigate whether increased gut permeability facilitates the migration of microbial products to the liver, triggering immune responses. Longitudinal studies could monitor gut permeability markers, such as ZO-1 and circulating sIgA, alongside LFT, such as ALT and as well as CRP levels, to identify patterns in KD progression. The potential relationship between increased gut permeability and liver dysfunction in KD is illustrated in **Figures 2A, B**.

## Gut permeability is linked to vasculitis and coronary artery lesions in Kawasaki disease

Cardiovascular disease (CVD) impacts various organs, notably the intestinal tract, often implicated early in the disease process (95). In CVD, heightened gut permeability is linked to systemic inflammation, potentially due to translocation of bacterial elements into the bloodstream and disruptions in immune responses within the gut (96). A study by Lai et al. demonstrated that serum levels of the TJ protein ZO-1 were lower in KD patients compared to controls, with these levels correlating to the formation of CAL (73). This suggests a possible connection between compromised gut permeability and CAL in KD. In KD, increased gut permeability is accompanied by elevated levels of circulating sIgA and IgA deposition in vascular tissues, as seen in a mouse model of KD vasculitis. Addressing gut barrier dysfunction in the model corrected permeability, prevented IgA deposition, and improved cardiovascular health. Furthermore, after LCWE injection in KD mice, gut permeability increased rapidly, preceding coronary artery inflammation and abdominal aorta dilation. These findings indicate that a compromised gut barrier may initiate rather than result from vasculitis. Treatment with AT1001, a zonulin inhibitor, effectively restored the gut integrity and reduced cardiovascular lesions, highlighting the importance of early gut barrier disruption in the development of KD vasculitis and coronary artery aneurysms (70).

The disruption of the gut barrier not only affects local inflammation but also influences broader immune mechanisms, including those involving key cytokines like IL-33, which are crucial for immune regulation and inflammation. The IL-33/ST2 axis plays a significant role in this context (97). IL-33, a cytokine from the IL-1 family, acts as an “alarm signal” when cells are damaged, being released primarily from the nuclei of endothelial and epithelial cells (98). It interacts with the ST2 receptor, which has two forms: transmembrane ST2 (ST2L), a membrane-bound receptor that triggers inflammatory responses when IL-33 binds to it; and soluble ST2 (sST2), a soluble form that can block the effects of IL-33 by acting as a decoy. This axis, therefore, helps regulate immune responses and inflammation (99). In the gut, IL-33 promotes the proliferation and differentiation of enterocytes, enhancing mucosal renewal and regeneration. It plays a pivotal role in balancing the Th1/Th2 immune response and regulating intestinal immune homeostasis (99). However, if the epithelial barrier is compromised, IL-33 release can trigger immune responses that worsen inflammation (100). During the acute phase of KD, serum levels of IL-33 and sST2—a decoy receptor that binds circulating IL-33—are significantly elevated (101, 102). Moreover, a recent study by Okada et al. found that KD patients, particularly those with CAL, had significantly elevated levels of sST2 during the acute phase. The study further demonstrated that IL-33 stimulates human coronary artery endothelial cells (HCAECs) leading to an upregulation of ST2L, which is necessary for the extracellular effects of IL-33. This stimulation also resulted in elevated production of sST2, IL-6, IL-8, and monocyte chemoattractant protein-1. These changes occurred in a time- and concentration-dependent manner, highlighting a proinflammatory role of IL-33. These findings suggest that the IL-33/ST2 axis plays a significant role in the pathogenesis of KD, particularly in the development of vasculitis and CAL (16). However, direct evidence linking increased gut permeability to the onset of systemic vasculitis and CAL in KD patients is still lacking. Further studies should aim to identify the specific mechanisms by which increased gut permeability contributes to the development of systemic vasculitis and CAL in KD. Key areas of investigation might include tracking changes in gut permeability over the course of KD progression and exploring the involvement of specific immune pathways, such as the IL-33/ST2 axis, in mediating the link between gut barrier disruption and systemic vasculitis. This targeted research could help clarify how early disruptions in gut integrity contribute to vascular complications, potentially leading to new strategies for early intervention and improved management of KD-related vasculitis. The hypothesis linking increased gut permeability to vasculitis and CAL in KD is summarized in **Figure 2C**.

## Gut permeability is linked to IVIG treatment in Kawasaki disease

IVIG therapy, the standard treatment for KD, primarily aims to reduce systemic inflammation and prevent complications such as CAL (5, 27). While its exact mechanisms remain complex, IVIG is thought to work by modulating the immune system (72). According to Nadig et al., IVIG can influence both the innate and adaptive immune responses. It affects the innate immune system by modifying pathways like TLR pathways, controlling autophagy and apoptosis in the mononuclear phagocytic system, managing the formation of neutrophil extracellular traps, and adjusting dendritic cell activity. In the adaptive immune system, IVIG helps regulate T-cell differentiation, cytokine production, and the function of Treg cells (103). Emerging evidence points to a potential connection between gut barrier function and IVIG efficacy. A multicenter study by Fabi et al., involving over 300 KD patients from 13 pediatric centers, evaluated the relationship between GI symptoms and the response to IVIG therapy. The study found that KD patients presenting with initial GI symptoms had a higher likelihood of IVIG unresponsiveness and a greater risk of developing coronary aneurysms. These findings suggest that GI involvement, possibly linked to gut permeability, could significantly influence the effectiveness of IVIG treatment and impact disease progression (3). Lai et al. observed that KD patients with CAL exhibited reduced serum levels of the TJ protein ZO-1. This suggests a potential link between compromised gut barrier function and the development of CAL in KD. However, the study did not find significant differences in ZO-1 levels when comparing IVIG-responsive and non-responsive patients, likely due to the study’s limited sample size (73). These findings suggest that increased gut permeability might contribute to IVIG unresponsiveness, but further large-scale studies are needed to confirm this connection. While it is not definitively established that IVIG directly stabilizes gut permeability, it is hypothesized that by reducing systemic inflammation—which can disrupt the gut barrier—IVIG might have an indirect stabilizing effect. Supporting this idea, Saito et al. found that IVIG treatment correlated with improvements in intestinal vascular permeability and mucosal damage caused by toxins from *Clostridium difficile* (104). However, studies directly comparing the levels of gut permeability markers before and after IVIG treatment in KD patients are limited. Future research should focus on understanding the role of gut permeability in KD and its influence on IVIG efficacy. This includes conducting larger, multicenter studies to validate the link between gut barrier dysfunction and IVIG responsiveness. Tracking markers like ZO-1 before and after IVIG treatment could clarify their predictive value for treatment outcomes.

## Gut permeability is linked to platelets in the pathogenesis of Kawasaki disease

PLT count is a crucial biomarker for monitoring KD progression and plays a significant role in assessing disease severity and guiding treatment decisions. Elevated PLT counts have been linked to increased risks of CAL, IVIG non-responsiveness, and KD shock syndrome (KDSS) (105–110). Specifically, patients with PLT counts greater than 450 × 10^9^/L at admission are at a higher risk of developing CAL (107). Park et al. conducted a study evaluating PLT counts in 505 KD patients during the acute phase, revealing that thrombocytosis—elevated PLT counts—was associated with worse clinical outcomes. Specifically, higher PLT counts correlated with prolonged hospital stays, longer fever durations, and a greater risk of IVIG non-responsiveness and coronary artery disease (108). Conversely, patients with post-hospitalization PLT counts below the normal range (<150 × 10^9^/L) have an increased risk of developing CAL. A rapid rise in PLT counts within the first 5 days of illness indicates heightened cytokine activity, which may damage the coronary arteries and lead to early-phase CAL (107). PLT counts are reported to decrease more significantly in patients with severe KD compared to others in the initial days of the illness (109, 110). Moreover, IVIG non-responding patients with the lowest PLT counts post-hospitalization are at a significantly increased risk of developing CAL independently (107). Before IVIG treatment, increased PLT aggregation and activation are observed, indicating early endothelial damage in KD (111, 112). This aggregation can lead to thrombocytopenia, prompting marrow stromal cells to produce more thrombopoietin, which enhances thrombopoiesis (113). In severe KD patients, thrombocytopenia may signal widespread vascular endothelium damage, causing further PLT aggregation and an increase in thrombopoiesis. Consequently, newly formed megakaryocytes and PLT have higher levels of vascular endothelial growth factor (VEGF) compared to mature PLT. These activated PLT cluster and release VEGF at sites of endothelial injury, perpetuating a vicious cycle that drives vascular inflammation and damage, contributing to the progression of CAL in KD (114). Moreover, activated PLT form complexes with monocytes and neutrophils—known as monocyte–PLT aggregates (MPAs) and neutrophil–PLT aggregates (NPAs)—which amplify the inflammatory response and increase IL-1β production, exacerbating vascular inflammation (9).

**Figure 3:**
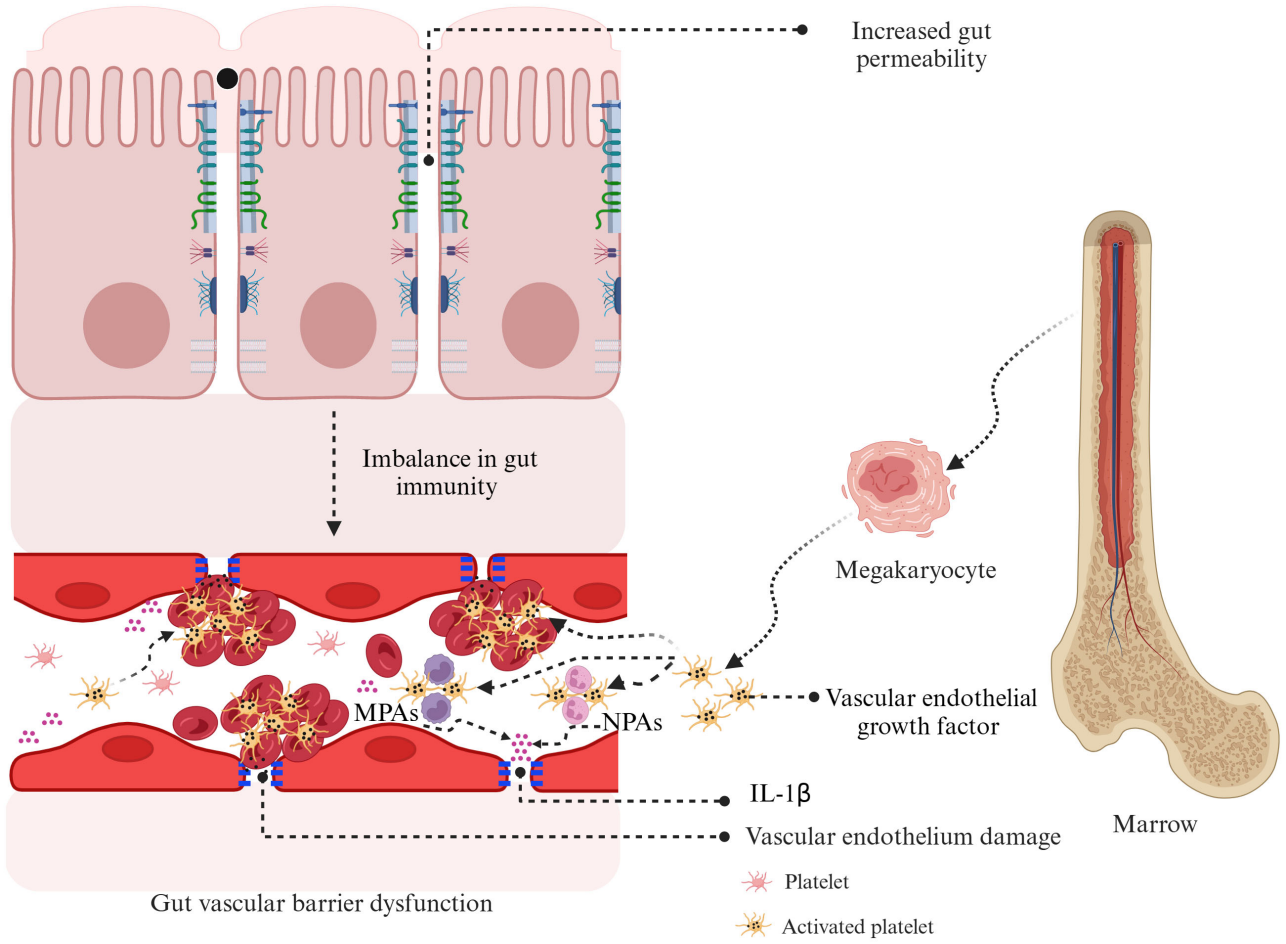
Hypothesized mechanisms linking gut permeability and platelet activation in Kawasaki disease. Increased gut permeability in KD can disrupt gut immunity and contribute to gut vascular barrier dysfunction. This dysfunction causes vascular endothelial damage, leading to the release of vascular endothelial growth factor from megakaryocytes and activated PLT. These events promote PLT aggregation, forming monocyte–PLT aggregates and neutrophil–PLT aggregates, which intensify inflammation through the production of interleukin-1β. This cycle of inflammation and vascular damage is thought to play a key role in the development of coronary artery lesions in KD. KD, Kawasaki disease; MPAs, monocyte–platelet aggregates; NPAs, neutrophil–platelet aggregates; IL-1β, interleukin-1β; PLT, platelet.

While the precise connection between gut permeability and PLT activation in KD remains unreported, recent research by Kocatürk et al. investigated PLT behavior in a KD animal model. The study showed that PLT aggregation contributes significantly to cardiovascular inflammation, primarily through the formation of MPAs. The study found that increased PLT counts and higher levels of MPAs were closely associated with severe cardiovascular inflammation. Notably, interventions that lowered PLT counts or inhibited PLT function effectively reduced inflammation, underscoring the critical role of PLT activation in worsening cardiovascular damage in KD vasculitis. This suggests that targeting MPAs, which enhance IL-1β production, could be a promising therapeutic strategy (9). Although this study did not specifically address the relationship between PLT counts and gut permeability, previous research using the same animal model demonstrated that gut permeability, alongside IgA deposition, plays a role in immune-mediated cardiovascular inflammation. Targeted interventions that restored gut barrier integrity not only corrected permeability issues but also prevented IgA buildup, leading to significant improvements in cardiovascular health (70). These findings suggest that both gut permeability and PLT activation are crucial factors in the development of vasculitis and cardiovascular complications in KD. It is hypothesized that increased gut permeability may disrupt gut immunity balance and damage the GVB, impacting vascular endothelial cells. This damage could promote PLT aggregation and activation, initiating a systemic inflammatory response, vascular inflammation, and the development of aneurysms. Understanding the link between gut permeability and PLT activation in KD is a priority for future research. Exploring this connection could shed light on KD’s pathogenesis and lead to innovative treatments. The potential mechanisms linking increased gut permeability to PLT activation in KD are illustrated in **Figure 3**.

## Future perspectives

As the complexities of KD continue to be unraveled, the GI tract’s significant role in its pathogenesis has become increasingly apparent. Emerging evidence linking increased gut permeability to PLT activation provides a novel avenue for understanding the pathogenesis of KD and its severe vascular complications, such as coronary artery aneurysms. This connection suggests that compromised gut barrier function may contribute to the systemic inflammation and vascular damage observed in KD. Future research should focus on delineating the exact mechanisms by which gut permeability contributes to KD pathogenesis. This could include longitudinal studies tracking changes in gut barrier integrity throughout the progression of KD and its responses to various treatments. Identifying specific pathways—such as alterations in TJ proteins, disruptions in microbial metabolites like SCFAs, and interactions between the gut microbiota and immune cells—will be crucial for a deeper understanding of how gut health influences systemic inflammation in KD. Interventions targeting the gut barrier offer promising strategies for reducing the severity of KD. Probiotics, prebiotics, and dietary modifications designed to restore gut microbiota balance could potentially reduce gut permeability, thereby decreasing systemic inflammatory responses and improving outcomes in KD patients. For example, probiotics like *C. butyricum* have shown potential in enhancing SCFAs production and reinforcing the gut barrier (1). Detailed evidence from these interventions can strengthen their applicability in KD management, potentially leading to more effective outcomes. A multidisciplinary approach is essential for a comprehensive understanding of KD. Gastroenterologists can lead efforts to optimize gut health through dietary and microbial interventions, while immunologists can explore immune pathways contributing to systemic inflammation. Cardiologists can assess the cardiovascular benefits of gut-targeted therapies. Collaborative clinical trials, integrated treatment guidelines, and shared research initiatives could pave the way for more holistic and effective KD management. The development of targeted therapies to strengthen the gut barrier or modulate the immune response at the gut level could also be transformative. Incorporating gut health into the routine clinical management of KD could lead to earlier diagnosis and more personalized treatment approaches. Understanding the relationship between the gut microbiota and KD may also help identify biomarkers for disease susceptibility and treatment efficacy. Ultimately, as research progresses, these insights are expected to provide a deeper understanding of the underlying mechanisms of KD, leading to improved clinical practices and better patient outcomes.
